# Docosahexaenoic Acid Stability in Ready-to-Use Therapeutic Food

**DOI:** 10.3390/foods12020308

**Published:** 2023-01-09

**Authors:** Genevieve James, Kevin Stephenson, Meghan Callaghan-Gillespie, Mohamed Tabita Kamara, Hui Gyu Park, J. Thomas Brenna, Mark J. Manary

**Affiliations:** 1 Dell Pediatric Research Institute, The University of Texas at Austin, Austin, TX 78723, USA; 2 Department of Medicine, Washington University, St. Louis, MO 63110, USA; 3 Department of Pediatrics, Washington University, St. Louis, MO 63110, USA; 4 Project Peanut Butter, Freetown 47235, Sierra Leone; 5 Children’s Nutrition Research Center, USDA-Agricultural Research Service, Houston, TX 77030, USA

**Keywords:** ready to use therapeutic food, DHA, omega-3, severe acute malnutrition, food processing

## Abstract

Ready-to-use therapeutic food (RUTF) is used to treat young children diagnosed with severe acute malnutrition. RUTF with low and balanced linoleic and alpha-linolenic acid, plus omega-3 docosahexaenoic acid (DHA), supports long-term cognitive recovery. DHA is prone to degradation due to peroxidation, possibly exacerbated by the iron inherently in RUTF. Our goals were to prepare benchtop and manufacturing scale of RUTF formulations that include DHA and measure its retention. Twenty-seven RUTF formulas with base ingredients, including oats, high oleic or commodity peanuts, and encapsulated or oil-based DHA at various levels were prepared at benchtop scale, followed by seven months of climate-controlled storage. These pilot samples had similar relative DHA retention. At the manufacturing scale, DHA was added at one of two stages in the process, either at the initial or the final mixing stage. Samples taken at preliminary or later steps show that less than 20% of DHA added at the early stages disappeared prior to packaging for any recipe tested. Overall, our data indicate that most DHA included in RUTF is retained in the final product and that DHA is best retained when added at the latest manufacturing stage.

## 1. Introduction

Over 50 million children under the age of five suffer from acute malnutrition globally, with approximately 16 million of these children having severe acute malnutrition (SAM) [1,2,3]. Ready-to-use therapeutic foods (RUTFs) are used on an outpatient basis to feed SAM children without additional medical complications, saving millions of lives [1]. In 2021, the first official international standard guidelines for RUTF formulation were finalized by the CODEX Committee on Nutrition and Foods for Special Dietary Uses (CCNFSDU) [4]. These guidelines are intended to provide a framework for formulating safe and efficacious RUTFs.

Children with SAM have increased energy needs for catch-up growth and anthropometric recovery. This drives relatively high lipid levels in RUTF. Linoleic acid (LA, omega-6) and alpha-linolenic acid (ALA, omega-3) are the only omega-6 and omega-3 fatty acids included in most RUTF formulations. The downstream omega-3 long-chain polyunsaturated fatty acid (LCPUFA), docosahexaenoic acid (DHA, omega-3), is required for brain development and must be synthesized endogenously from ALA when not consumed directly from the diet. Endogenous conversion of ALA to DHA is inefficient, producing small amounts of DHA [5]. LA and ALA are converted to downstream LCPUFA by the same core set of enzymes, and excess LA reduces ALA conversion. This has been demonstrated in RUTF, where Standard formulations with high LA cause a precipitous drop in circulating DHA [6,7]. This can be remedied by a better balance between dietary LA and ALA. Our recent clinical trial shows that children consuming RUTF with balanced LA: ALA and preformed DHA achieve long-term improvement in neurodevelopment compared to those who consume RUTF with high LA: ALA and no DHA [8].

The new guidelines set the recommended maximum value for omega-6 fatty acids at 780 mg/100 kcal, and a minimum value of 110 mg/100 kcal for omega-3 fatty acids (Table 1). Reformulating RUTFs to comply with the new guidelines and support cognition in children with SAM is straightforward, with a judicious choice of ingredients. However, DHA is a novel ingredient in RUTF manufacturing and is known to be oxidatively labile. Here, we report formulations conforming to the new global CODEX guidelines on LA and ALA, while also including DHA. We formulated 27 different recipes and measured DHA levels following about seven months’ storage in a climate-controlled setting. Additionally, we produced a series of samples where DHA was added at different points in the RUTF manufacturing process and evaluated its retention in the final product. Taken together, these results will provide insight into DHA stability in RUTF formulations during the manufacturing process and after storage.

## 2. Materials and Methods

### 2.1. DHA Materials

The four forms of DHA were used in these experiments are described in Table 2. The DHA sources were donated by their producers in vacuum-sealed foil envelopes with the stability of several months at temperatures <25 °C. Package integrity and storage temperatures <25 °C were maintained during the study.

### 2.2. RUTF Essential Fatty Acid Reformulation

Standard RUTF is typically composed of peanuts, skimmed milk powder, sugar, and vegetable oil in similar proportions as well as 3% of concentrated vitamin and mineral powder and 2% of hydrogenated vegetable stabilizer to prevent excessive oil separation. The most common vegetable oils used are soy oil, palm oil, sunflower oil, and canola oil. The total oil content of RUTF must be >28% to create a low-moisture, semisolid food that can be swallowed by infants in substantial quantities. Thus, RUTF usually has high amounts of omega-6 and low amounts of omega-3. To comply with the recently approved CODEX guidelines, reductions in omega-6 and increases in omega-3 are needed. Using nutrient profiles for candidate ingredients that were found on the USDA’s FoodData Central, a food and nutrient database [9], the omega-6 and omega-3 content of common and potential RUTF ingredients were identified (Supplementary Table S1).

Substitutions in the types of vegetable oil and peanuts used in RUTF can achieve sufficient reductions in omega-6, particularly LA. For standard RUTF recipes, peanuts are the major contributor to omega-6 as a percentage of total energy. Switching standard peanuts for a high-oleic (HO) variety significantly reduces the LA percentage of total energy (example in Supplementary Table S2) [6,7]. The LA: ALA ratio can be further improved by using oils with lower amounts of LA and/or higher amounts of ALA, such as canola oil. Additionally, depending on local availability, legumes and cereals (e.g., chickpeas, lentils, oats) can potentially be used to reduce or replace peanuts and other ingredients, such as oil stabilizers in RUTFs. RUTF, made with oats, offers an example of an acceptable alternative formulation that can use a reduced amount of HO peanuts and does not require a hydrogenated vegetable oil stabilizer. A typical RUTF oat formulation contains 17% peanut, 18% oat, 18.5% skimmed milk, 5.5% canola oil, 12.9% palm oil, 17% sugar, 8.2% whey permeate, and 2.9% micronutrient premix.

Increasing omega-3 content sufficiently to comply with the new CODEX guidelines solely by increasing ALA can be difficult since doing so requires incorporating specialty edible oils. Fish oil offers a viable commercial food additive that readily provides omega-3 with the added benefit of supporting cognition in children [8,10].

Adding preformed DHA into RUTF formulas requires the consideration of several factors, including the ideal form to use (oil vs. powder), encapsulation ingredients, cost, stability/shelf life, and incorporation into the RUTF production process. The most common form of preformed DHA is an omega-3 PUFA fish oil concentrate. This is a challenging form to incorporate into food matrices due to its higher susceptibility to oxidation and rancidity. While there are handling techniques and ways to stabilize omega-3 fish oil concentrates, we determined that powdered forms produced by spray drying are a superior alternative. The microencapsulation technologies stabilize omega-3 fish oil against oxidization and thus help keep fish oil stable for the shelf-life of the food product. Studies have confirmed that microencapsulated fish oil is bioavailable and bioequivalent to dietary supplementation with soft-gel capsules [11,12]. Multiple encapsulation source materials/options (e.g., sucrose, fish, bovine dairy) are commercially available to satisfy different dietary practices (e.g., vegetarian, kosher) (Supplementary Table S3).

### 2.3. DHA Stability in Stored RUTF

Twenty-seven RUTF recipes with added DHA were prepared using a standard benchtop method [13] and held in climate-controlled conditions for 7 months. All recipes were viable therapeutic alternatives, incorporating 75–100 mg of DHA per 100 g of RUTF (Supplementary Table S4A,B). Three spray-dried DHA powders (Table 2A–C) and one fish oil concentrate (Table 2D) were chosen. The powders contain tuna oil in a microencapsulated form and are our preferred form of DHA for RUTF recipes. The fish oil concentrate was chosen for comparison of organoleptic properties. RUTF formulations were prepared at the benchtop level in a food lab at Washington University in St. Louis and were evaluated for their mixing and flow properties. Slight adjustments were made to the formulations and prepared in the food lab.

### 2.4. DHA Stability in the RUTF Manufacturing Process

**Figure 1:**
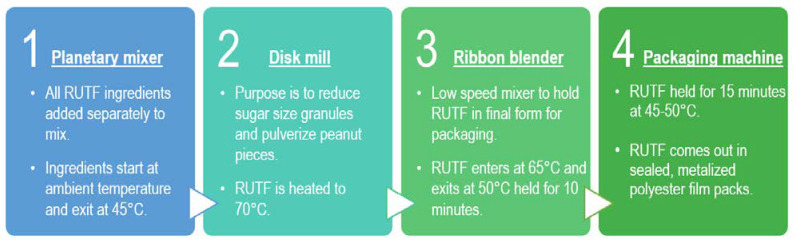
RUTF Manufacturing Process Stages. Samples were taken from stages 1 and 4 for the DHA stability tests. All ingredients are mixed in the planetary mixer before entering the disk mill, where the remaining larger pieces are pulverized to create a homogenous mixture. The ribbon blender mixes the RUTF at low speed and prepares it for packaging in stage 4. Temperatures range from 45–70 °C between the end of the planetary mixer and the packaging machine phase.

Two RUTF formulations were used to evaluate DHA stability in the manufacturing process, one with HO peanuts (Peanut) and the other containing oats and HO peanuts (Oat–Peanut) (Supplementary Table S5). DHA’s vulnerability to heat, light, and oxygen makes DHA loss a potential concern, as production temperatures can range between 45 °C and 70 °C. Additionally, the presence of lipid oxidizing metal catalysts, such as iron and copper, might increase losses. To test DHA stability in RUTF manufacturing, a series of tests were run, wherein DHA was added in the planetary mixer or the packaging machine phase (Figure 1). Samples were then taken from both the planetary mixer and the packaging machine; these samples were stored in conical polypropylene tubes or metalized PET sachets, respectively. All samples were stored in climate-controlled conditions prior to laboratory analysis.

### 2.5. Fatty Acid Analysis

Fatty acid methyl esters (FAME) were prepared from each sample utilizing a one-step digestion, transmethylation, and FAME extraction procedure modified from that described previously [14,15]. The FAME were dissolved into heptane and injected into a GC-flame ionization detector for quantification according to previously reported methods [8]. Results are reported as a percentage by weight of total fat.

### 2.6. Statistical Analysis

Statistical analyses were performed using Microsoft Office Excel 2022. To evaluate DHA stability in stored RUTF, least squares regression was used to compare the amounts of DHA added versus the DHA measured. To evaluate DHA stability in the RUTF manufacturing process, *t*-tests were used to calculate the statistical significance of differences in fatty acids between groups. Differences were considered significant when *p* < 0.05.

## 3. Results

**Figure 2:**
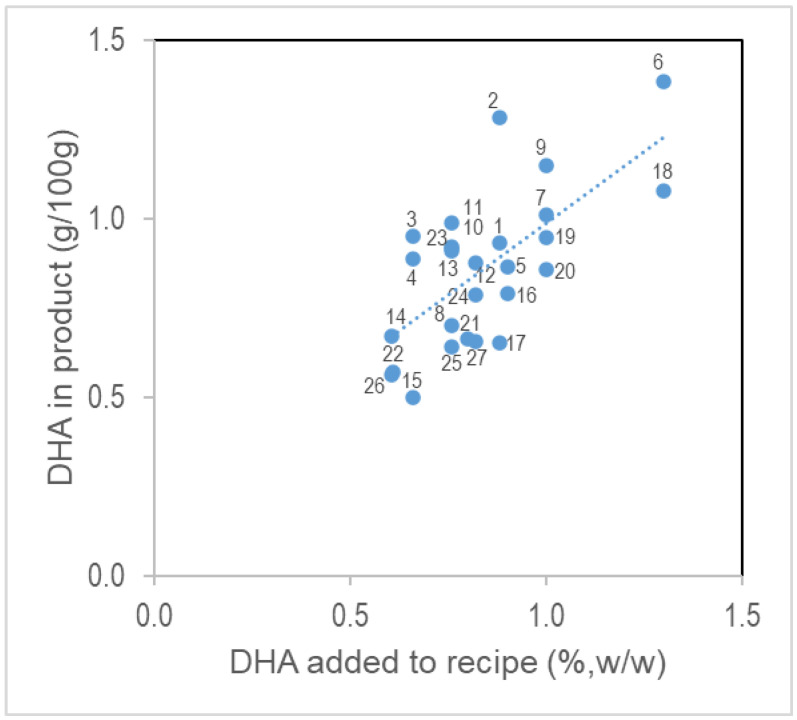
DHA in RUTF. The amount of DHA added to RUTF is linearly related to the amount added. Recipe ingredients for each formulation are in Supplementary Table S4A,B.

Analysis of the 27 benchtop RUTF formulations showed a strong linear correlation between the amount of DHA added to the recipe and the amount of DHA measured in the RUTF (*p* < 0.05, Figure 2). When the time of recipe storage came, a form of DHA (encapsulated or unencapsulated fish oil), and the presence or absence of oat flour were included in a linear regression model, none of these covariate coefficients were significant.

For the oat–peanut formula, DHA comprised 0.24% w/w of the fatty acid profile in the sample taken from the planetary mixer and dropped to 0.19% w/w in the sample taken from the packaging machine (Supplementary Table S6: the 0.05% w/w decrease corresponds to a 20% apparent loss in DHA (*p* = 6.9 × 10^−4^). DHA in the peanut formula comprised 0.27% (w/w) of the fatty acid profile in both types of samples taken (n.s., *p* = 0.36). No major differences were found among non-DHA fatty acids.

**Figure 3:**
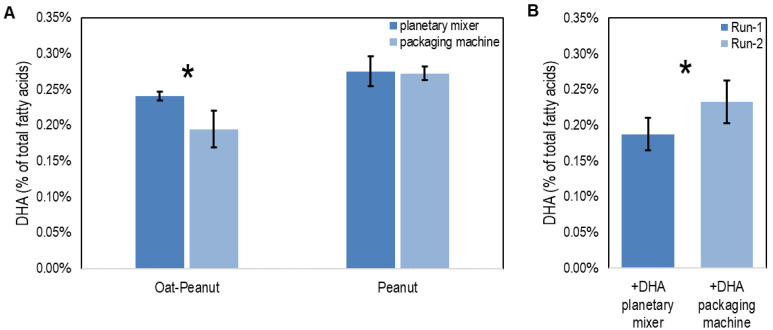
DHA stability in the RUTF manufacturing process. (**A**) DHA stability in the oat–peanut and peanut formulas at two points in the RUTF manufacturing process. DHA was added in the initial planetary mixer phase. Samples were taken from the planetary mixer (*n* = 5) and packaging machine (*n* = 5) phases. The means for the oat–peanut formulation are significantly different, indicating DHA loss when it is added to the planetary mixer. The means for the peanut formulation were not different. (**B**) Comparing DHA stability in the peanut formula when DHA is added at different points in the manufacturing process. In run-1, DHA was added in the planetary mixer phase. In run-2, DHA was added in the packaging machine phase. Samples were taken from the packaging machine phase for both runs (*n* = 5). The means are significantly different between run-1 and run-2, indicating there is DHA loss when it is added in the planetary mixer instead of the packaging machine. (**A**,**B**) MEG-3™ DHA rf Powder (B, Table 2) was the DHA source for all formulations. Errors bars indicate mean ± SD, % by weight of total fat. *T*-tests were done to calculate statistical significance (* *p* < 0.05).

Additional testing was conducted on the peanut formula since its formulation most closely resembles the typical RUTF, with the main difference being HO peanuts. In this test, DHA was added at the beginning (Run-1, planetary mixer) or the end of the manufacturing process (Run-2, packaging machine). DHA levels were stable in Run-1, comprising 0.17% (w/w) of the fatty acid profile in samples taken from the planetary mixer and 0.19% in samples taken from the packaging machine (Supplementary Table S7) (n.s., *p* = 0.32). In Run-2, DHA was added at the packaging machine stage and comprised 0.23% of the fatty acid profile. Comparing samples taken from the packaging machine for Run-1 (DHA at planetary mixer) and Run-2 (DHA added at packaging machine), DHA levels decreased by 0.04% w/w, representing roughly a 17% decrease in the weight of DHA in the final product (Figure 3B) (*p* = 0.02).

## 4. Discussion

RUTFs are a key component in the medical and dietary management of SAM, and they have saved the lives of millions of children with SAM [2]. The new CODEX RUTF formulation guidelines require decreasing the maximum omega-6 and increasing the minimum omega-3 content to better support recovery from SAM. The fatty acid profile of traditional and many existing RUTF formulations do not support omega-3 LCPUFA status; these PUFAs have well-established effects on long-term neurodevelopment in preclinical studies [16]. Older RUTF formulas also have high LA content, extreme LA:ALA ratios, and no preformed DHA. DHA is present in breast milk of normally-nourished women and in omnivorous diets [17]. Additionally, formulations with excess LA lead to a dramatic deterioration of omega-3 LCPUFA status [6,18,19].

LA is an essential fatty acid required for skin integrity and as a precursor for key signaling eicosanoids. LA deficiency manifests in skin lesions, polydipsia, and faltering growth [20]. Dietary levels of 1–2% energy fully support skin integrity and growth [21]. However, excess LA competes with ALA for the enzymes needed to endogenously produce omega-3 LCPUFA, such as eicosapentaenoic acid (EPA, omega-3) and DHA [22,23]. Current standard RUTFs have LA content in excess of 7.9% of total energy and minimal ALA, resulting in LA: ALA ratios between approximately 11.3:1 and higher [7,20,24]. These dietary factors suppress endogenous omega-3 LCPUFA synthesis and inhibit their incorporation into tissues [6,7,19]. Simply increasing ALA content improves EPA levels but does not improve or maintain DHA levels if high dietary LA intake is not also decreased [5].

Reformulating RUTFs to have lower LA content and a balanced LA: ALA ratio requires careful consideration of ingredients and their contribution to the total fatty acid profile. We recommend starting RUTF reformulation with a survey on what ingredients contribute to the fatty acid profile, paying particular attention to their contribution to LA and ALA content. Once fatty-acid-contributing ingredients are identified, one can compile a list of suitable ingredients for reducing LA content and/or improving the LA: ALA ratio. It may be helpful to emphasize ingredients that are more locally accessible and economically feasible. Peanuts are the most used base ingredient in RUTFs and are the highest contributor to LA content. Switching from conventional to HO peanuts substantially reduces LA. Moreover, foods and oils with increased oleic acid (OA) substituting, and thus, decreasing LA content, are more stable and less prone to lipid oxidation, improving shelf-life and organoleptic properties [7,25]. Using oils with a sizable amount of ALA, such as canola oil, instead of oils with little or no ALA can also improve the LA: ALA ratio. The new CODEX guidelines allow for LA: ALA ratios as high as 7:1, but RUTFs with lower LA:ALA ratios better support omega-3 LCPUFA nutritional status [6,7,22]. Apart from these changes, the ingredients used here are established as RUTF components and, thus, raise no concerns with respect to limiting the shelf-life of the final product.

One option for complying with the new recommended omega-3 minimum level is to increase the ALA level. However, edible oils high in ALA can be difficult to source. A commercial food additive alternative that may be easier to acquire is fish oil, which has the added advantage of providing significant amounts of omega-3 in the form of EPA and DHA. Apart from being present in human breast milk, dietary preformed DHA is included in 99% of infant formulas in the U.S., in part based on compelling preclinical and clinical evidence directed to neurodevelopment, especially in pregnancy through the first two years of life and likely, well into childhood [26,27,28]. Children with SAM consuming RUTF as nearly the sole source of calories will have tissue fatty acid profiles defined by the fatty acid profile of the RUTF and modified by genetics [22]. Thus, fatty acid proportions are particularly critical as the brain is restoring integrity and function with the influx of calories and protein [16,29,30]. RUTF with decreased LA and a 4:1 LA: ALA ratio is sufficient for the maintenance of DHA nutritional status and significantly improves EPA status [6,19]. However, only the addition of preformed DHA in RUTF formulas has been shown to significantly improve DHA status and enhance neurocognition in children with SAM [8,19]. The weight of preclinical and clinical evidence strongly supports the inclusion of DHA in RUTF.

Liquid fish oil concentrate is the most common form of preformed DHA. Encapsulated DHA powders are widely available, appear to have more shelf-life stability due to oxidation resistance, and may be simpler to add to the RUTF during manufacture. RUTF contains minerals, including iron, which may catalyze the oxidation of DHA, particularly at elevated temperatures. We found that encapsulated and unencapsulated fish oil can be added to the RUTF at multiple points without obvious odors. However, our findings indicate that preformed DHA is best added as late as possible in the manufacturing process to minimize DHA loss. Adding DHA earlier resulted in 17–20% lower DHA in the product. Once sealed in the final package (sachet), our results indicate that DHA remains stable in storage. Finally, it should be noted that the rheological behavior of encapsulated DHA is similar to dry particles such as sugar or milk powder. Therefore, it cannot be counted as part of the necessary 28% to create a liquid needed to feed young children.

There were several limitations to this study. For evaluation of DHA stability in the RUTF manufacturing process, DHA levels were only measured in recipes containing an encapsulated form of DHA. Recipes with an unencapsulated form of DHA, such as fish oil concentrate, may perform differently. Although we expect that encapsulated DHA would be more stable to oxidation than a liquid oil containing DHA, we did not test oil and therefore cannot speculate as to whether any differences in DHA loss and stability would be significant. Additionally, DHA loss during manufacturing was only evaluated in two different recipe types (Oat–Peanut and Peanut), so loss estimates may not be applicable to all RUTF formulations. The formulations also had increased OA, which is more stable and resistant to oxidation, so it may have conferred some protection against DHA loss. It is possible that formulations with lower amounts of OA could exhibit increased DHA loss.

## 5. Conclusions

Our data show numerous RUTF recipes that include and preserve DHA during storage in the final product. Losses no greater than 20% were observed even when DHA was added at an early stage in the RUTF manufacturing process. DHA losses were minimal when added later. As DHA sources are relatively expensive compared to other components, the addition late in the process is the most economically efficient for achieving a target DHA level in the final product. Once incorporated and sealed in the final product, DHA levels in RUTF remain stable in room-temperature storage.

## Figures and Tables

**Table 1:** Ready-to-use therapeutic foods fatty acid composition guidelines ^a^.

Nutrient	% Total Energy	kcal/100 g	g/100 g	mg/100 kcal
Energy		520–550		
Lipids	45–60	234–330	26–36.7	5–7
n-6 fatty acids	2.9–6.7	15.3–36.9	1.7–4.1	330–780
n-3 fatty acids	1–2.5	5.4–13.5	0.6–1.5	110–280

^a^ Adapted from guidelines agreed upon in the 45th session of the Joint Food and Agriculture Organization/World Health Organization Food Standards Programme, CODEX Alimentarius Commission [4].

**Table 2:** DHA sources used in RUTF formulations.

Code	Encapsulation Source Material	Source of Formulation Ingredients	Allergens Beyond Fish	Storage and Stability	Product
A	Sucrose	modified food starch, mixed natural tocopherols, sucrose	mixed tocopherols	Sensitive to air, heat, light, and humidity. May be stored for 24 months from the date of manufacture in an unopened original container (which is sealed under inert gas) and at temperatures below 25 °C (77 °F). Once open use contents quickly.	MEG-3^®^ ‘15’ n-3 High DHA Powder S/SD
B	Fish gelatin	sunflower oil, mixed natural tocopherals	mixed tocopherols, soybean oil	Unopened packages in refrigerated conditions 36–46 °F for up to 18 months. Open packages in refrigerated conditions up to 1 week.	MEG-3™ DHA rf Powder
C	Dairy	sodium caseinate, dextrose monohydrate, dried glucose syrup	contains milk, soy, and fish	May be stored for 24 months from the data of manufacture in the unopened original package in dry, cool conditions (10–25 °C). After opening bag, the gas headspace should be flushed with inert gas prior to being resealed. Contents of resealed bag should be used within 3 months after resealing.	Nu-Mega Driphorm HiDHA 50
D	Fish oil Concentrate	concentrated wild Alaskan pollock oil omega-3 triglyceride, natural mixed tocopherols	mixed tocopherols	May be stored for three years from the date of manufacture in an unopened original container (which is sealed under inert gas) and at temperatures below 25 °C (77 °F). Once open use contents quickly.	AlaskOmega TG 230460

## Data Availability

Data available upon request from Corresponding Author.

## Supplementary Materials

The following supporting information can be downloaded at: https://www.mdpi.com/article/10.3390/foods12020308/s1, Table S1: Energy (kcal) and fatty acid composition (% total FA) of RUTF ingredients ranked from lowest to highest LA value; Table S2: Fatty acid profile comparison between identical recipes using high oleic or non-high oleic peanuts; Table S3: Examples of commercially available DHA sources; Table S4A: Oat-Peanut benchtop formulations used to assess DHA stability in storage; Table S4B: Peanut benchtop formulations used to assess DHA stability in storage; Table S5: Formulations used to assess DHA stability in the RUTF manufacturing process; Table S6: DHA stability in the oat-peanut and peanut formulas at two points in the RUTF manufacturing process; Table S7: Comparing DHA stability in the peanut formula when DHA is added at different points in the manufacturing process.

## References

[1] Lanyero B., Namusoke H., Nabukeera-Barungi N., Grenov B., Mupere E., Michaelsen K.F., Mølgaard C., Christensen V.B., Friis H., Briend A. (2017). Transition from F-75 to ready-to-use therapeutic food in children with severe acute malnutrition, an observational study in Uganda. Nutr. J..

[2] UNICEF, WHO, World Bank Group (2019). Levels and Trends in Child Malnutrition: Key Findings of the 2019 Edition; UNICEF, WHO, and the World Bank Group Joint Child Malnutrition Estimates. https://www.who.int/publications/i/item/WHO-NMH-NHD-19.20.

[3] Moustiés C., Bourlieu-Lacanal C., Hemery Y.M., Baréa B., Villeneuve P., Servent A., Alter P., Lebrun M., Laillou A., Wieringa F.T. (2022). Nutritional quality of ready-to-use therapeutic foods: Focus on lipid composition and vitamin content. OCL.

[4] (2021). Report of the 42nd Session of the Codex Committee on Nutrition and Foods for Special Dietary Uses. https://www.usda.gov/sites/default/files/documents/ccnfsdu-delegates-report.pdf.

[5] Brenna J.T., Salem N., Sinclair A.J., Cunnane S.C. (2009). α-Linolenic acid supplementation and conversion to n-3 long-chain polyunsaturated fatty acids in humans. Prostaglandins Leukot. Essent. Fat. Acids.

[6] Hsieh J.-C., Liu L., Zeilani M., Ickes S., Trehan I., Maleta K., Craig C., Thakwalakwa C., Singh L., Brenna J.T. (2015). High-oleic ready-to-use therapeutic food maintains docosahexaenoic acid status in severe malnutrition. J. Pediatr. Gastroenterol. Nutr..

[7] Brenna J.T., Akomo P., Bahwere P., Berkley J.A., Calder P.C., Jones K.D., Liu L., Manary M., Trehan I., Briend A. (2015). Balancing omega-6 and omega-3 fatty acids in ready-to-use therapeutic foods (RUTF). BMC Med..

[8] Stephenson K., Callaghan-Gillespie M., Maleta K., Nkhoma M., George M., Park H.G., Lee R., Humphries-Cuff I., Lacombe R.J.S., Wegner D.R. (2022). Low linoleic acid foods with added DHA given to Malawian children with severe acute malnutrition improve cognition: A randomized, triple-blinded, controlled clinical trial. Am. J. Clin. Nutr..

[9] United States Department of Agriculture (USDA) FoodData Central. https://fdc.nal.usda.gov/.

[10] Sittiprapaporn P., Bumrungpert A., Suyajai P., Stough C. (2022). Effectiveness of fish Oil-DHA supplementation for cognitive function in Thai children: A randomized, doubled-blind, two-dose, placebo-controlled clinical trial. Foods.

[11] Wallace J.M., McCabe A.J., Robson P.J., Keogh M.K., Murray C.A., Kelly P.M., Márquez-Ruiz G., McGlynn H., Gilmore W.S., Strain J.J. (2000). Bioavailability of n-3 polyunsaturated fatty acids (PUFA) in foods enriched with microencapsulated fish oil. Ann. Nutr. Metab..

[12] Sanguansri L., Augustin M.A., Lockett T.J., Abeywardena M.Y., Royle P.J., Mano M.T., Patten G.S. (2015). Bioequivalence of n-3 fatty acids from microencapsulated fish oil formulations in human subjects. Br. J. Nutr..

[13] Zuzarte A., Mui M., Ordiz M.I., Weber J., Ryan K., Manary M.J. (2020). Reducing oil separation in ready-to-use therapeutic food. Foods.

[14] Garces R., Mancha M. (1993). One-step lipid extraction and fatty acid methyl esters preparation from fresh plant tissues. Anal. Biochem..

[15] Zhou Y., Nijland M., Miller M., Ford S., Nathanielsz P.W., Brenna J.T. (2008). The influence of maternal early to mid-gestation nutrient restriction on long chain polyunsaturated fatty acids in fetal sheep. Lipids.

[16] Brenna J.T. (2011). Animal studies of the functional consequences of suboptimal polyunsaturated fatty acid status during pregnancy, lactation and early post-natal life. Matern. Child Nutr..

[17] Brenna J.T., Varamini B., Jensen R.G., Diersen-Schade D.A., Boettcher J.A., Arterburn L.M. (2007). Docosahexaenoic and arachidonic acid concentrations in human breast milk worldwide. Am. J. Clin. Nutr..

[18] Babirekere-Iriso E., Mortensen C.G., Mupere E., Rytter M.J.H., Namusoke H., Michaelsen K.F., Briend A., Stark K.D., Friis H., Lauritzen L. (2016). Changes in whole-blood PUFA and their predictors during recovery from severe acute malnutrition. Br. J. Nutr..

[19] Jones K.D., Ali R., Khasira M.A., Odera D., West A.L., Koster G., Akomo P., Talbert A.W., Goss V.M., Ngari M. (2015). Ready-to-use therapeutic food with elevated n-3 polyunsaturated fatty acid content, with or without fish oil, to treat severe acute malnutrition: A randomized controlled trial. BMC Med..

[20] Holman R.T. (1998). The slow discovery of the importance of omega 3 essential fatty acids in human health. J. Nutr..

[21] Holman R.T. (1971). Biological activities of and requirements for polyunsaturated acids. Prog. Chem. Fats Other Lipids.

[22] Zhang J.Y., Kothapalli K.S., Brenna J.T. (2016). Desaturase and elongase-limiting endogenous long-chain polyunsaturated fatty acid biosynthesis. Curr. Opin. Clin. Nutr. Metab. Care.

[23] Lattka E., Illig T., Koletzko B., Heinrich J. (2010). Genetic variants of the FADS1 FADS2 gene cluster as related to essential fatty acid metabolism. Curr. Opin. Lipidol..

[24] Hibbeln J.R., Nieminen L.R., Blasbalg T.L., Riggs J.A., Lands W.E. (2006). Healthy intakes of n-3 and n-6 fatty acids: Estimations considering worldwide diversity. Am. J. Clin. Nutr..

[25] Riveros C.G., Mestrallet M.G., Gayol M.F., Quiroga P.R., Nepote V., Grosso N.R. (2010). Effect of storage on chemical and sensory profiles of peanut pastes prepared with high-oleic and normal peanuts. J. Sci. Food Agric..

[26] Lauritzen L., Brambilla P., Mazzocchi A., Harsløf L.B., Ciappolino V., Agostoni C. (2016). DHA effects in brain development and function. Nutrients.

[27] Martinez M. (1992). Tissue levels of polyunsaturated fatty acids during early human development. J. Pediatr..

[28] Brenna J.T. (2016). Long-chain polyunsaturated fatty acids and the preterm infant: A case study in developmentally sensitive nutrient needs in the United States1–4. Am. J. Clin. Nutr..

[29] Marín M.C., Rey G.E., Pedersolí L.C., Rodrigo M.A., de Alaniz M.J. (2000). Dietary long-chain fatty acids and visual response in malnourished nursing infants. Prostaglandins Leukot Essent Fat. Acids.

[30] Lelijveld N., Jalloh A.A., Kampondeni S.D., Seal A., Wells J.C., Goyheneix M., Chimwezi E., Mallewa M., Nyirenda M.J., Heyderman R.S. (2019). Brain MRI and cognitive function seven years after surviving an episode of severe acute malnutrition in a cohort of Malawian children. Public Health Nutr..

