# Supplementary material for: Docosahexaenoic Acid Stability in Ready-to-Use Therapeutic Food
Source: Foods. 2023 Jan 9;12(2):308. doi: 10.3390/foods12020308 (PMC9858440; doi:10.3390/foods12020308)
Supplement: Supplementary file 1 [file foods-12-00308-s001.zip › foods-2105412-supplementary.pdf]

Supplementary Materials

Docosahexaenoic acid stability in ready-to-use therapeutic food

Genevieve James,[1] Kevin Stephenson,[2] Meghan Callaghan-Gillespie,[3] Mohamed Tabita Kamara,[4] Hui Gyu Park,[1] J. Thomas Brenna,[1] and Mark J. Manary[3,4,5]

[1]Dell Pediatric Research Institute, University of Texas at Austin, Austin, TX 78723, USA; [2]Department of Medicine, Washington University, St. Louis, MO 63110, USA; [3]Department of Pediatrics, Washington University, St. Louis, MO 63110, USA; [4]Project Peanut Butter Sierra Leone, Freetown; [5]USDA/Agricultural Research Service Children's Nutrition Research Center, Houston, TX, USA

Supplementary Table 1. Energy (kcal) and fatty acid composition (% total FA) of RUTF ingredients ranked from lowest to highest LA value.

Oils	Total energy	Total fat	Total MUFA	Total PUFA	Total n-6 PUFA	Total n-3 PUFA	LA	ALA	LA:ALA
Palm kernel	862	100	11.4	1.6	1.6	0.0	**1.6**	0.0	UD
Coconut	892	99.1	6.3	1.7	1.7	0.0	**1.7**	0.0	88.2
Sunflower (high oleic, 70% and over)	884	100	83.7	3.8	3.6	0.2	**3.6**	0.2	18.8
Peanut (high oleic, Texas)	NR	100	82.7	3.7	3.7	NR	**3.7**	NR	NR
Palm	884	100	37.0	9.3	9.1	0.2	**9.1**	0.2	45.5
Safflower (high oleic, oil of commerce)	884	100	75.2	12.8	12.7	0.1	**12.7**	0.1	132.5
Canola (high oleic)	900	100	72.7	15.8	13.1	2.2	**12.9**	2.2	5.9
Flax (cold pressed)	884	100	18.4	67.8	14.3	53.4	**14.2**	53.4	0.3
Canola (high oleic, >70%, Natreon)	884	100	72.0	17.1	14.9	2.1	**14.5**	2.1	7.0
Almond	884	100	69.9	17.4	17.4	0.0	**17.4**	0.0	UD
Perilla (Korea)	NR	100	12.9	78.2	NR	NR	**18.3**	59.9	0.3
Canola (rapeseed)	884	100	63.3	28.1	18.6	9.1	**18.6**	9.1	2.0
Sunflower (mid-oleic)	884	100	57.3	29.0	28.7	0.0	**28.7**	0.0	775.8
Peanut	884	100	46.2	32.0	32.0	0.0	**32.0**	0.0	UD
Sunflower (linoleic <60%)	884	100	45.4	40.1	39.8	0.2	**39.8**	0.2	199.0
Sesame	884	100	39.7	41.7	41.3	0.3	**41.3**	0.3	137.7
Soy	884	100	22.8	57.7	50.4	6.8	**50.4**	6.8	7.4
Peanut (high oleic, refined)	857	100	71.4	17.9	NR	NR	**NR**	NR	NR
Peanut (high oleic, unrefined)	857	100	78.6	7.1	NR	NR	**NR**	NR	NR

NR = not reported or calculated if LA or ALA information was missing; UD = undefined, typically high levels LA and negligible ALA

Supplementary Table 2. Fatty acid profile comparison between identical recipes using high oleic or non-high oleic peanuts[a]

Common name	Fatty acid	Oat-Peanut 1A[b]	Oat-Peanut 1B[c]
Lauric acid	12:0	0.06	0.13
Lauroleic acid	12:1	0.08	0.12
Myristic acid	14:0	0.55	0.26
Myristoleic acid	14:1	0.58	0.25
Palmitic acid	16:0	21.89	24.38
Palmitoleic acid	16:1	0.27	0.19
Stearic acid	18:0	2.58	3.02
Oleic acid	18:1	58.66	50.29
Linoleic acid	18:2n-6	11.07	16.63
α-Linolenic acid	18:3n-3	1.82	1.88
Arachidic acid	20:0	0.44	0.55
Eicosenoic acid	20:1	0.85	0.51
Arachidonic acid	20:4n-6	0.01	0.02
Behenic acid	22:0	0.58	0.76
Erucic acid	22:1	0.03	0.00
Eicosapentaenoic acid	20:5n-3	0.06	0.05
Docosadienoic acid	22:2	0.26	0.34
Adrenic acid	22:4n-6	0.11	0.13
Lignoceric acid	24:0	0.40	0.45
Nervonic acid	24:1	0.03	0.02
Docosapentaenoic acid	22:5n-3	0.01	0.01
Docosahexaenoic acid	22:6n-3	0.23[b]	0.26

[a] Content expressed in grams per 100 grams
[b] Formulated with high-oleic peanuts
[c] Formulated with conventional peanuts

This table shows the fatty acid profile of identical Oat-Peanut recipes (Oat-Peanut recipe ingredients in Supplementary Table 4). Oat-Peanut 1A was made with high-oleic peanuts and Oat-Peanut 1B was made with conventional peanuts. Using high-oleic peanuts resulted in a 33.4% decrease in linoleic acid, a 14.3% increase in oleic acid, and an improved LA:ALA ratio going from 8.8:1 to 6.1:1.

Supplementary Table 3. Examples of commercially available DHA sources

Code	Encapsulation source material	Source of formulation ingredients	Allergens beyond fish	Storage and stability	Product
A	Sucrose	modified food starch, mixed natural tocopherols, sucrose	mixed tocopherols	Sensitive to air, heat, light and humidity. May be stored for 24 months from the date of manufacture in an unopened original container (which is sealed under inert gas) and at temperatures below 25°C (77°F). Once open use contents quickly.	MEG-3® '15' n-3 High DHA Powder S/SD*
B	Fish gelatin	sunflower oil, mixed natural tocopherals	mixed tocopherols, soybean oil	Unopened packages in refrigerated conditions 36-46°F for up to 18 months. Open packages in refrigerated conditions up to 1 week.	MEG-3™ DHA rf Powder
C	Dairy	sodium caseinate, dextrose monohydrate, dried glucose syrup	contains milk, soy and fish	May be stored for 24 months from the data of manufacture in the unopened original package in dry, cool, conditions (10-25°C). After opening bag, the gas headspace should be flushed with inert gas prior to being resealed. Contents of resealed bag should be used within 3 months after resealing.	Nu-Mega Driphorm HiDHA 50
D	Fish oil Concentrate	concentrated wild Alaskan pollock oil omega-3 triglyceride, natural mixed tocopherols	mixed tocopherols	May be stored for three years from the date of manufacture in an unopened original container (which is sealed under inert gas) and at temperatures below 25°C (77°F). Once open use contents quickly.	AlaskOmega TG 230460
E	Bovine	canola oil, sunflower oil, mixed natural tocopherols (E 306)	mixed natual tocopherols extracted from soybeans	Unopened packages in refrigerated conditions 36-46°F up to 18 months. Open packages in refrigerated conditions up to 1 week.	MEG-3® DHA B Powder
F	Fish gelatin	canola oil, sunflower oil, mixed natural tocopherols (E 306)	mixed natual tocopherols extracted from soybeans	Unopened packages in refrigerated conditions 36-46°F up to 18 months. Open packages in refrigerated conditions up to 1 week.	MEG-3® DHA K Powder
G	Sucrose	corn syrup solids, starch	contains soy and fish	May be stored for 24 months from the data of manufacture in the unopened original package in dry, cool, conditions (10-25°C). After opening bag, the gas headspace should be flushed with inert gas prior to being resealed. Contents of resealed bag should be used within 3 months after resealing.	Nu-Mega Driphorm HiDHA 30
H	Dairy	sodium caseinate, dextrose monohydrate, dried glucose syrup	contains milk, soy and fish	May be stored for 24 months from the data of manufacture in the unopened original package in dry, cool, conditions (10-25°C). After opening bag, the gas headspace should be flushed with inert gas prior to being resealed- contents of resealed bag should be used within 3 months after resealing.	Nu-Mega Driphorm HiDHA 60

Supplementary Table 4A. Oat-Peanut benchtop formulations used to assess DHA stability in storage[a]

Ingredient (in grams)	Oat-Peanut Formulations													
	1[b]	2[b]	3[b]	4[b]	5[b]	6[b]	7[b]	8[c]	9[c]	10[c]	11[d]	12[d]	13[c]	14[d]
Oats	18.0	18.0	17.5	17.9	18.0	17.5	17.0	18.0	17.0	18.0	18.0	18.0	18.0	18.0
High oleic peanuts	17.1	16.9	17.0	17.0	17.0	16.5	16.5	16.5	16.5	17.0	16.5	16.5	16.5	16.5
Dry skim milk	18.6	18.6	18.7	18.5	18.5	19.0	19.0	18.5	19.0	18.5	19.0	19.0	18.5	19.0
Whey Permeate	7.8	8.8	8.6	8.3	8.2	8.0	9.6	7.3	7.6	7.4	7.4	7.4	7.3	7.4
Canola Oil	6.1	6.0	5.9	5.8	5.2	6.0	6.0	6.0	6.0	5.5	6.0	6.0	6.0	6.0
Palm Oil	12.9	12.2	12.7	12.6	12.6	13.0	13.0	13.0	13.0	12.9	13.0	13.0	13.0	13.0
Sugar (white)	15.7	15.7	16.0	16.3	16.7	15.8	15.0	17.0	17.0	17.0	17.0	17.0	17.0	17.0
Premix	2.9	2.9	2.9	2.9	2.9	2.9	2.9	2.9	2.9	2.9	2.9	2.9	2.9	2.9
Preformed DHA	0.9	0.9	0.7	0.7	0.9	1.4	1.0	0.8	1.0	0.8	0.23	0.2	0.76	0.17

[a] Content expressed per 100 grams
[b] MEG-3® '15' n-3 High DHA Powder S/SD (A, **Table 3**)
[c] MEG-3™ DHA rf Powder (B, **Table 3**)
[d] AlaskOmega TG 230460 (D, **Table 3**)

Supplementary Table 4B. Peanut benchtop formulations used to assess DHA stability in storage[a]

Ingredient (in grams)	Peanut Formulations												
	15[b]	16[b]	17[b]	18[b]	19[b]	20[c]	21[c]	22[c]	23[c]	24[d]	25[c]	26[d]	27[d]
High oleic peanuts	24.0	24.1	24.0	24.0	24.0	24.0	24.0	24.0	24.0	24.3	24.0	24.0	24.0
Dry skim milk	26.0	25.5	26.0	26.0	25.5	26.0	25.7	25.9	26.0	25.5	26.0	26.0	26.0
Whey Permeate	8.3	8.6	8.1	8.6	9.2	8.9	9.2	9.2	10.2	9.4	8.2	8.8	8.7
Canola Oil	6.0	6.0	6.0	6.0	6.0	6.0	6.0	6.0	6.0	6.0	6.0	6.0	6.0
Palm Oil	12.1	11.3	12.1	11.2	11.4	11.2	11.4	11.4	11.2	11.6	11.2	12.0	12.0
Sugar (white)	18.0	18.7	18.0	18.0	18.0	18.0	18.0	18.0	19.0	18.0	19.0	18.2	18.2
Premix	2.9	2.9	2.9	2.0	2.0	2.9	2.9	2.9	2.9	2.9	2.9	2.9	2.9
Emulsifier	2.0	2.0	2.0	2.9	2.9	2.0	2.0	2.0	0.0	2.0	2.0	2.0	2.0
Preformed DHA	0.7	0.9	0.9	1.3	1.0	1.0	0.8	0.6	0.8	0.23	0.8	0.2	0.2

[a] Content expressed per 100 grams
[b] MEG-3® '15' n-3 High DHA Powder S/SD (A, **Table 3**)
[c] MEG-3™ DHA rf Powder (B, **Table 3**)
[d] AlaskOmega TG 230460 (D, **Table 3**)

Supplementary Table 5. Formulations used to assess DHA stability in the RUTF manufacturing process[a]

Ingredient	Oat-Peanut[b]	Peanut[b]
Oat, g	18.0	0.0
High oleic peanuts, g	17.0	24.0
Dry skim milk, g	18.5	26.0
Whey Permeate, g	7.4	10.2
Canola Oil, g	5.5	6.0
Palm Oil, g	12.9	11.2
Sugar (white), g	17.0	19.0
Premix, g	2.9	2.9
Emulsifier, g	0.0	0.0
Preformed DHA, g	0.76	0.76

[a] Content expressed per 100 grams
[b] MEG-3™ DHA rf Powder (B, Table 2) is the preformed DHA source

Supplementary Table 6. DHA stability in the oat-peanut and peanut formulas at two points in the RUTF manufacturing process[a]

Fatty acid	Oat-Peanut[b] (PM)	Oat-Peanut[c] (PaM)	Peanut[b] (PM)	Peanut[c] (PaM)
12:0	0.1 ± 0.05	0.14 ± 0.01	0.07 ± 0.01	0.08 ± 0.03
12:1	1.83 ± 0.9	2.26 ± 1.49	0.73 ± 0.35	1.07 ± 1.06
14:0	0.34 ± 0.04	0.31 ± 0.05	0.31 ± 0.06	0.24 ± 0.01
14:1	1.25 ± 0.89	5.55 ± 0.88	0.23 ± 0.05	2.19 ± 1.16
16:0	21.23 ± 0.29	20.83 ± 0.87	18.06 ± 0.37	18.53 ± 0.34
16:1	0.16 ± 0.02	0.16 ± 0	0.13 ± 0	0.16 ± 0.02
18:0	2.92 ± 0.04	2.8 ± 0.09	2.75 ± 0.01	2.69 ± 0.06
18:1	56.48 ± 0.57	53.33 ± 1.17	62.86 ± 0.48	60.34 ± 1.44
18:2n-6	11.11 ± 0.22	10.34 ± 0.36	9.48 ± 0.12	9.65 ± 0.21
18:3n-3	1.74 ± 0.05	1.75 ± 0.56	1.84 ± 0.16	1.77 ± 0.03
20:0	0.35 ± 0.02	0.32 ± 0.01	0.44 ± 0.01	0.37 ± 0.03
20:1	0.8 ± 0.02	0.72 ± 0.03	1.09 ± 0.02	0.97 ± 0.06
20:4n-6	0.02 ± 0	0.01 ± 0	0.02 ± 0	0.02 ± 0
22:0	0.5 ± 0.03	0.44 ± 0.04	0.76 ± 0.02	0.66 ± 0.04
22:1	0.12 ± 0	0.09 ± 0.03	0.13 ± 0	0.12 ± 0
20:5n-3	0.06 ± 0.01	0.04 ± 0.01	0.06 ± 0.01	0.06 ± 0
22:2	0.07 ± 0.02	0.06 ± 0.01	0.05 ± 0.02	0.05 ± 0.01
22:4n-6	0.29 ± 0.05	0.29 ± 0.06	0.13 ± 0.03	0.23 ± 0.09
24:0	0.4 ± 0.01	0.35 ± 0.03	0.59 ± 0.02	0.52 ± 0.03
22:6n-3	0.24 ± 0.01	0.19 ± 0.02	0.27 ± 0.03	0.27 ± 0.01

[a] Results are expressed as a percentage by weight of total fat. Mean ± SD. DHA powder was the preformed DHA source for both formulations (MEG-3™ DHA rf Powder, B in Table 2). PM, sample taken from the planetary mixer; PaM, sample taken from the packaging machine.
[b] DHA was added during the planetary mixer phase
[c] DHA was added during the packaging phase

Supplementary Table 7. Comparing DHA stability in the peanut formula when DHA is added at different points in the manufacturing process[a]

Fatty acid	Peanut-1[b] (PM)	Peanut-1[b] (PaM)	Peanut-2[c] (PM)	Peanut-2[c] (PaM)
12:0	0.11 ± 0.02	0.11 ± 0.02	0.14 ± 0.01	0.12 ± 0.01
12:1	2.19 ± 2.31	2.92 ± 3.92	7.06 ± 2.51	4.05 ± 2.06
14:0	0.43 ± 0.05	0.28 ± 0.05	0.28 ± 0.07	0.25 ± 0.05
14:1	1.01 ± 0.86	0.74 ± 0.71	0.9 ± 0.3	0.68 ± 0.3
16:0	26.52 ± 0.62	23.23 ± 0.94	25.16 ± 0.71	23.01 ± 0.45
16:1	0.17 ± 0.02	0.15 ± 0.02	0.13 ± 0	0.15 ± 0.02
18:0	3.62 ± 0.1	3.35 ± 0.17	3.5 ± 0.1	3.34 ± 0.09
18:1	54.62 ± 2.14	57.27 ± 3.01	51.72 ± 2	56.69 ± 1.57
18:2n-6	8.84 ± 0.28	8.55 ± 0.45	8.43 ± 0.26	8.23 ± 0.23
18:3n-3	0.72 ± 0.07	0.76 ± 0.1	0.76 ± 0.03	0.69 ± 0.05
20:0	0.28 ± 0.05	0.31 ± 0.06	0.2 ± 0.03	0.29 ± 0.04
20:1	0.55 ± 0.08	0.66 ± 0.35	0.62 ± 0.04	0.83 ± 0.05
20:4n-6	0.02 ± 0.01	0.02 ± 0	0 ± 0	0.02 ± 0
22:0	0.31 ± 0.07	0.6 ± 0.06	0.44 ± 0.04	0.61 ± 0.03
22:1	0.04 ± 0.01	0.07 ± 0.01	0.07 ± 0	0.07 ± 0.01
20:5n-3	0.05 ± 0.01	0.05 ± 0	0 ± 0	0.06 ± 0.01
22:2	0.03 ± 0	0.04 ± 0.01	0.03 ± 0.01	0.03 ± 0.01
22:4n-6	0.14 ± 0.07	0.29 ± 0.25	0.26 ± 0.05	0.2 ± 0.05
24:0	0.17 ± 0.05	0.43 ± 0.07	0.32 ± 0.03	0.45 ± 0.03
22:6n-3	0.17 ± 0.04	0.19 ± 0.02	0 ± 0	0.23 ± 0.03

[a] Results are expressed as a percentage by weight of total fat. Mean ± SD. DHA powder was the preformed DHA source for both formulations (MEG-3™ DHA rf Powder, B in Table 2) PM, sample taken from the planetary mixer; PaM, sample taken from the packaging machine.

[b] DHA was added during the planetary mixer phase

[c] DHA was added during the packaging machine phase
